# C-951-07. Prospective Randomized Controlled Trial of Fresh Frozen Plasma versus Albumin in Acute Burn Resuscitation

**DOI:** 10.1093/jbcr/irag033.115

**Published:** 2026-04-06

**Authors:** Arek J Wiktor, Scott W Mueller, Blaire Balstad, Thomas O Vogler, Elena Shergina, Kathryn Colborn, Munib Far, Justin Burleson, Cameron Gibson, Alexandra Halevi, Fred Endorf, Adit A Ginde

**Affiliations:** Burn & Frostbite Center at UCHealth, University of Colorado Hospital, Aurora, CO; Burn & Frostbite Center at UCHealth, University of Colorado Hospital, Aurora, CO; University of Colorado Hospital, Aurora, CO; University of Colorado, Aurora, CO; University of Colorado Anschutz, School of Medicine, Aurora, CO; University of Colorado Anschutz, School of Medicine, Aurora, CO; UCHealth/University of Colorado Hospital, Aurora, CO; UCHealth/University of Colorado Hospital, Aurora, CO; University of Colorado, Aurora, CO; University of Colorado Anschutz, School of Medicine, Aurora, CO; University of Colorado, Aurora, CO; University of Colorado Anschutz, School of Medicine, Aurora, CO

## Abstract

**Introduction:**

There is no consensus between use of fresh frozen plasma (FFP) and albumin (ALB) during acute burn resuscitation. The comparative impact on vascular endotheliopathy (ENDO) is also unknown. Our objective was to determine the efficacy and safety of FFP versus ALB during burn resuscitation. We hypothesize that FFP will reduce 48 hour total fluid volumes and correct burn ENDO better than ALB.

**Methods:**

This non-blinded prospective randomized controlled trial of acute thermal burn patients occurred at an ABA-verified burn center. Patients with ≥20% TBSA burn admitted within 8 hours (hrs) post injury were eligible. Those with trauma requiring transfusion, stage IV/V kidney disease, or expected death within 48 hrs post burn were excluded. Enrolled patients were resuscitated using a Nurse Driven Resuscitation Protocol with lactated ringers and received either FFP or 5% ALB at 8 hrs post burn at a total volume of 0.5 ml x kg x %TBSA over 8 hrs. Demographics, severity of injury, 48 hr fluids, and complications for 1 week post-burn were recorded. Products of ENDO were collected on admit, 12, 24, and 48 hrs. Data are presented as mean (±standard deviation) or median [interquartile range] depending on normality. A t-test (two-sided α = 0.05) was used to compare the primary outcome, 24 hr total volume of fluids.

**Results:**

Of 124 patients screened, 59 were enrolled. One was excluded for receiving both colloids during the 8 hour intervention window. Patients were well matched at baseline between FFP and ALB: age 46 (±15) vs 42 (±15), male 25 (86%) vs 19 (65%), %TBSA 28 [24-44] vs 37 [23-45], mechanical ventilation 14 (48%) vs 15 (52%), inhalation injury (4 each), burn injury time to admit 4.4 (±2) vs 3.9 (±2) hrs, and vasopressor use 13 (45%) vs 12 (41%), respectively. Total fluid volume at 24 hrs post burn (ml/kg/%TBSA) was not different between FFP 5.4 (±2.2) and ALB 4.7 (±1.6), *p*=.14, nor at 25-48 hrs FFP 2.2 (±1.2) and ALB 2.5 (± 1.2), *p*=.3. Delta lactate (peak - 24 hrs) was similar FFP -2 (±2.4) and ALB -2.1 (±3). Thirteen complications (3 ARDS, 1 renal replacement therapy, 1 acute kidney injury, 8 pulmonary edema) occurred in 9 FFP patients, whereas 20 (3 ARDS, 2 renal replacement therapy, 4 acute kidney injury, 11 pulmonary edema) occurred in 11 ALB patients. There were no abdominal compartment syndromes. Hospital mortality occurred in 4 (14%) FFP and 3 (10%) ALB patients.

**Conclusions:**

No difference was observed in total fluid volumes administered, complications, or mortality between FFP and ALB in acute burn resuscitation. Further research is needed to address patient specific differences, and optimal colloid dosing and timing for burn resuscitation. ENDO analysis is pending.

**Applicability of Research to Practice:**

Fresh frozen plasma and albumin appear similar in regards to clinical efficacy and safety for acute burn resuscitation.

**Funding for the study:**

Military Burn Research Program Clinical Translational Research Award.

